# Aptly chosen, effectively emphasizing the action and mechanism of antimycin A_1_

**DOI:** 10.3389/fmicb.2024.1371850

**Published:** 2024-04-03

**Authors:** Linyan Zhu, Chenhong Weng, Xiaoman Shen, Xiangdong Zhu

**Affiliations:** ^1^School of Life Science and Biopharmaceutics, Shenyang Pharmaceutical University, Shenyang, China; ^2^College of Biological Science and Engineering, Jiangxi Agricultural University, Nanchang, Jiangxi, China

**Keywords:** Antimycin A_1_, *Rhizoctonia solani*, inhibition mechanism, mitochondrial complex III and IV, genes in mitochondria, metabolomics analysis

## Abstract

*Rhizoctonia solani* Kühn, a plant pathogenic fungus that can cause diseases in multiple plant species is considered one of the common and destructive pathogens in many crops. This study investigated the action of antimycin A_1,_ which was isolated from *Streptomyces* AHF-20 found in the rhizosphere soil of an ancient banyan tree, on *Rhizoctonia solani* and its mechanism. The inhibitory effect of antimycin A_1_ on *R. solani* was assessed using the comparative growth rate method. The results revealed that antimycin A_1_ exhibited a 92.55% inhibition rate against *R. solani* at a concentration of 26.66 μg/mL, with an EC_50_ value of 1.25 μg/mL. To observe the impact of antimycin A_1_ on mycelial morphology and ultrastructure, the fungal mycelium was treated with 6.66 μg/mL antimycin A_1_, and scanning electron microscopy (SEM) and transmission electron microscopy (TEM) were employed. SEM analysis demonstrated that antimycin A_1_ caused mycelial morphology to become stripped, rough, and folded. The mycelium experienced severe distortion and breakage, with incomplete or locally enlarged ends, shortened branches, and reduced numbers. TEM observation revealed thickened cell walls, indistinct organelle boundaries, swollen mitochondria, exosmotic substances in vesicles, slow vesicle fusion, and cavitation. Real-time quantitative PCR and enzyme activity assays were conducted to further investigate the impact of antimycin A_1_ on mitochondria. The physiological and biochemical results indicated that antimycin A_1_ inhibited complexes III and IV of the mitochondrial electron transport chain. RT-PCR analysis demonstrated that antimycin A_1_ controlled the synthesis of relevant enzymes by suppressing the transcription levels of ATP6, ATP8, COX3, QCR6, CytB, ND1, and ND3 genes in mitochondria. Additionally, a metabolomic analysis revealed that antimycin A_1_ significantly impacted 12 metabolic pathways. These pathways likely experienced alterations in their metabolite profiles due to the inhibitory effects of antimycin A_1_. Consequently, the findings of this research contribute to the potential development of novel fungicides.

## Introduction

*Rhizoctonia solani* Kühn is a plant pathogenic fungus that can cause diseases in multiple plant species. It is considered one of the common and destructive pathogens in many crops. This pathogen has a wide host range, infecting over 32 families and 188 genera of plants, including cereals, legumes, vegetables, and several economically important crops (Taguchi-Shiobara et al., 2013). Rice sheath blight caused by the fungus *Rhizoctonia solani* is one of the most devastating rice diseases in the world (Bhaskar et al., 2020). This filamentous fungus affects a wide range of cultivated crops, and that its pathogenic mechanisms may vary among different plant hosts. The fungus attacks the rice at all stages of its growth and leads to seed rot, seedling sheath blight and death (Abdalla and Abdel-Fattah, 2000). It is responsible for causing stem rot symptoms in tomatoes, hypocotyl and root rot symptoms in spinach, broccoli, and melons, as well as bottom rot in lettuce (Eiko et al., 2003).

The most widely used method for controlling the plant diseases is the use of fungicides. This plant disease has been controlled by spraying validamycin and other antibiotics, and the high persistence of antibiotics and their harmful effects on non-target organisms and humans represents a major challenge (Molla et al., 2020). The development of resistance to fungicides has also become a concern. Therefore, people are searching for molecules with safer fungicidal activity to control this plant disease (Yadav et al., 2007). The use of microorganisms from soil and rhizosphere to control plant diseases has been in practice for a long time; over hundreds of millions of years of evolution, microorganisms have produced highly complex secondary metabolite molecules with diverse structures and rich three-dimensional structures. The discovery of natural fungicides has sparked significant interest among people.

Researchers have conducted studies on antimicrobial microorganisms against plant pathogens and discovered that a substantial proportion of the active compounds are produced by *Streptomyces* species. These microorganisms produce approximately 100,000 antibiotic compounds, which account for 70–80% of all naturally occurring bioactive products with pharmaceutical or agrochemical applications (Bubici, 2018; Abdel-Razek et al., 2020). Among them, the antifungal compounds known as antimycins are the most important class, with 46 naturally occurring antimycin compounds discovered to date.

The molecular structure of antimycin compounds is composed of a unique nine-membered bicyclic lactone core connected to a 3-methylamino salicylamide through an amide bond. The structural differences among various derivatives mainly lie in the substituent groups at positions C-7 and C-8 of the core. These compounds exhibit various biological activities. In 2005, Shiomi et al. (2005) evaluated the antimicrobial activities of antimycin A_9_ against *Saccharomyces cerevisiae*, *Shizosaccharomyces* pombe, *Aspergillus niger*, *Mucor racemosus*, *Penicillium chrysogenum* and *Trichophyton mentagrophytes*, with the most significant activity observed against *Trichophyton mentagrophytes*, with a minimum inhibitory concentration (MIC) of 0.31 μg/mL, but the values against other microorganismswere >10 mg/mL. Hosotani et al. (2005) assessed the activity of antimycin A_10_ ~ A_16_ and antimycin A_3_ against *Candida utilis* NBRC10707 using a paper disc assay, where antimycin A_3_ exhibited the largest inhibition zone diameter. They also found that hydroxylation at the C-8 position significantly increased the antifungal activity, while the length of the alkyl side chain at C-7 and the acyl side chain at C-8 were negatively correlated with the antifungal activity of antimycins. Antimycins specifically bind to the cytochrome bc1 complex on the inner mitochondrial membrane, disrupting the transfer of electrons from cytochrome b to cytochrome c1, thus inhibiting respiration (Strangman et al., 2009). Antimycins also bind to the mitochondrial membrane protein Bcl-2, and this specific binding disrupts the stability of the mitochondrial membrane potential, leading to cell apoptosis (Miyoshi et al., 1995).

In the pursuit of alternatives to agricultural chemicals, research has demonstrated the effectiveness of microbial secondary metabolism in managing rice diseases and enhancing rice growth and yield. Considering the significance of this pathogen and previous endeavors to combat the disease using biological agents, the objective of this study was to utilize the secondary metabolism of microorganisms to control the pathogen. In the early stage of this project, we isolated *Streptomyces* AHF-20 from the rhizosphere soil of an ancient banyan tree planted during the Wanli period of Ming Dynasty (AD 1572–1,620) in Jian City, Jiangxi Province, China, which had broad-spectrum antibacterial ability (Li et al., 2019), and an active secondary metabolism ingredient antimycin A_1_ was isolated and characteried from its fermentation broth, which could inhibit the growth of *Rhizoctonia solani*. Streptomyces species have long been regarded as a valuable source of natural products due to their extensive and intricate secondary metabolism (Genilloud, 2017; Xia et al., 2020; Chang et al., 2021). These results prompted us to further research the antimicrobial action of the secondary metabolism of *Streptomyces* AHF-20 against *R. solani*. There have been limited studies on the mechanism of action of antimycin A_1_ against *R. solani*. However, it has been suggested that antimycin A_1_ could potentially inhibit *R. solani* by suppressing the respiratory chain and disrupting the mitochondrial membrane. Therefore, the findings of this research contribute to the potential development of novel fungicides. The study highlights antimycin A_1_ as a potent inhibitor of the fungus *Rhizoctonia solani*, providing valuable insights into its inhibitory mechanism and impact on fungal morphology, ultrastructure, and mitochondrial function. Understanding the mode of action of antimycin A_1_ can serve as a basis for the development of new fungicides or the optimization of existing ones that target similar pathways or mechanisms.

## Materials and methods

### Materials

Antimycin A_1_ (95%) was obtained from Shanghai Maokang Biotechnology Co., Ltd. All chemicals used were of analytical grade. Scanning electron microscopy (SEM, HT7800) and transmission electron microscopy (TEM, SU8100) were used to characterize the mycelial morphology and ultrastructure of *R. solani*. The CheKine™ Micro Mitochondrial Complex III and IV Activity Assay Kit was purchased from Abbkine Scientific Co., Ltd., Wuhan, China.

### Culture conditions

*Rhizoctonia solani* Kȕhn used in the experiment was provided by the Laboratory of College of Agronomy, Jiangxi Agricultural University.

PDA (Potato Dextrose Agar Medium): 200 g of potatoes were peeled and 800 mL of distilled water was added and boiled for 15–30 min. The boiled water was filtered carefully with multilayer gauze to obtain clear solution, then 20 g of glucose and 20 g of agar powder were added. Then, water was added to get 1 L of solution before the solution was transferred to a high-pressure steam sterilization pot and sterilized at 121°C for 25 min. PDA was then air-dried on a superclean workbench at approximately 60°C and poured into a sterile petri dish. The solidified material was used for fungal inoculation and natural pH.

### *Rhizoctonia solani* activation

Fresh fungal mycelium cake (5 mm) was taken from the edge of the colony, inoculated onto PDA solid medium, and cultured at 28°C for 3 days. The activated fungi were obtained by continuous transfer for 2 generations.

### Anti-fungal activity of antimycin A_1_ against *Rhizoctonia solani*

The inhibitory effect of different concentrations of antimycin A_1_ on the mycelial growth of *R. solani* was determined by the mycelial growth rate method (Yang et al., 2020). The specific methods were as follows. Antimycin A_1_ was dissolved in a small amount of DMSO (dimethyl sulfoxide), prepared in a 1 mg/mL antimycin A_1_ solution with sterilized distilled water in a sterile environment and mixed with PDA medium. PDA medium with final concentrations of 0.416 μg/mL, 0.83 μg/mL, 1.66 μg/mL, 3.33 μg/mL, 6.66 μg/mL, 13.33 μg/mL and 26.66 μg/mL with DMSO concentrations less than 0.25% was prepared in a sterile workbench. In the centre of the plate, *R. solani* mycelium cake (5 mm) was inserted, the mycelia were facing down, the petri dish was sealed with a sealing film, and a plate containing an equal amount of sterile water with a DMSO concentration less than 0.25% was used as a blank control. Each treatment was repeated 3 times, and the culture was carried out in an incubator at 25°C. In all groups, the colony diameter of each plate was measured by the cross-crossing method, the colony growth inhibition rate of antimycin A_1_ against *R. solani* was calculated, and the average inhibitory rate was taken. The following formula was used to calculate:

$$\mathrm{Inhibition}\;\mathrm{rate}\%=\left[\frac{\left(C-d\right)-\left(S-d\right)}{\left(C-d\right)}\right]\times 100\%$$

C and S are the average diameters of fungal colonies in the blank control group and the antimycin A_1_ treatment group, respectively, and d is the diameter of the fungal cake.

### Measurement of TTC-DRA, protein leakage and K^+^ leakage

Dehydrogenase activity was determined using the method based on the Erdogan Eliuz study (Erdoğan Eliuz, 2021). Colourless TTC accepted H during cellular respiration and produced insoluble red-coloured 2,3,5-triphenylformazan (TF). The reduction amount of TTC was converted to determine the dehydrogenase activity. The absorbance of TF molecules was measured at 485 nm to determine the activity of dehydrogenase in the cells after adding antimycin A_1_ into the culture medium of *R. solani*.

A test tube containing 10 mL of liquid potato medium was incubated at 180 rpm and 25°C for 4 h. Then, antimycin A_1_ was added to final concentrations of 6.66 μg/mL and 13.33 μg/mL, and the culture was continued at 25°C and 180 rpm for 2 h. Distilled water was used as a blank control, and validamycin was used as a positive control. Fifty microlitres of the fungal mixture cultured for 2 h as described above and 50 μL TTC-glucose solution were added to a 96-well plate, and the mixture was shaken at 100 rpm in a shaking incubator for 30 min to form the red substance TF. Finally, the dehydrogenase activity was measured by absorbance at 485 nm using an automatic microplate reader. Dehydrogenase activity (%) = (ODx/ODc) × 100, where ODx and ODc represent the absorbance of treated and control samples, respectively.

Detection of protein leakage was performed as follows: 50 μL of the mixture of *R. solani* and antimycin A_1_ was transferred into a 96-well plate, 50 μL of Coomassie brilliant blue dye reagent was added to the 96-well plate for staining for 5 min, and colour was developed in the 96-well plate. Intracellular protein leakage was determined using the method based on the Erdogan Eliuz study (Erdoğan Eliuz, 2021). The absorbance was measured at 595 nm with an automatic microplate reader, and the protein leakage rate in the culture medium treated with antimycin A_1_ was calculated.

Protein leakage rate (%) = (ODx − ODc)/ODc × 100, where ODx and ODc represent the absorbance of the sample group treated with antimycin A_1_ and the blank control group, respectively.

Detection of K^+^ leakage was performed as follows. The KNO_3_ standard solution was prepared, 5 mL of the abovementioned mixture of *R. solani* treated with antimycin A_1_ was centrifuged at 10000 × g for 5 min, 0.5 mL of the supernatant was placed in a 50 mL centrifuge tube, 4.5 mL of caesium chloride solution (5 g/L) was added, and then 45 mL of deionized water was added again. The solution was thoroughly mixed, and the absorption value was determined by an atomic absorption spectrophotometer.

### Testing the conductivity

*Rhizoctonia solani* was activated on an aseptic PDA medium plate. When the hyphae grew to cover the whole plate, two pieces of hyphal cakes with a diameter of 5 mm were taken with a professional hole puncher, inoculated into 50 mL PDA liquid medium, and cultured in an oscillating chamber at 25°C and 180 rpm for 3 days. Cakes were removed to retain the mycelium, and the culture medium on the surface of the mycelium was removed and rinsed with deionized water more than 3 times. Then, 1 g of the above mycelia was weighed and added to antimycin A_1_ solution prepared with sterile distilled water at a concentration of 6.66 μg/mL and 13.33 μg/mL to be set as the treatment group and distilled water as the blank control group, with 3 replicates per concentration. The mixture was placed on a constant temperature oscillator at 25°C and 180 rpm for 4 h and then centrifuged at 3500 rpm for 5 min. The supernatant was removed, and the conductivity change of the culture medium treated with antimycin A_1_ was tested with a conductivity meter (Yu et al., 2023). The electrode was rinsed with deionized water before detection.

### The morphology and ultrastructure of *Rhizoctonia solani* mycelia

The morphology and ultrastructure of *R. solani* were examined using scanning electron microscopy (SEM) and transmission electron microscopy (TEM) techniques, respectively. Antimycin A_1_ was added to the PDA plate until the final concentration was 6.66 μg/mL. A mycelial cake of *R. solani* with a diameter of 5 mm was inoculated on a PDA plate with antimycin A_1_ and cultured at 25°C for 72 h. A piece of mycelia (4 mm × 4 mm) was sliced and immersed in 2.5% glutaraldehyde at 4°C for 12 h, followed by fixation in 1% OsO4 at 4°C for 2 h and dehydration through a graded ethanol series. After critical point drying, the samples were sputter-coated and observed using a scanning electron microscope (Hitachi, S4800, Japan). Other R. solani mycelia treated with antimycin A_1_ was embedded in resin, cured at 37°C for 12 h, and then polymerized at 60°C for 48 h. Thin sections were cut, double-stained with uranyl acetate and lead citrate, and observed using a HT7700 transmission electron microscope (HITACHI) (Zhang et al., 2018).

### Effects of antimycin A_1_ on the mitochondrial complex III − IV of *Rhizoctonia solani*

#### Mitochondrial extraction

The mitochondria were extracted using the protocol provided by the mitochondrion isolation kit (BioVision, U.S.), and the protein concentration of isolated mitochondria were determined using BCA kit (Solarbio, China).

1 The collected mycelia treated with and without antimycin A_1_ were washed with PBS and put into a mortar. Ten millilitres of liquid nitrogen was added for full grinding. Tissue (0.1 g) was accurately weighed, and 1 mL of reagent I and 10 μL of reagent III were added to the centrifuge tube.

2 The mixture was centrifuged at 600 × g for 5 min at 4°C, the supernatant was collected and poured into another new centrifuge tube, and the precipitate was discarded.

3 The supernatant was centrifuged again at 4°C and 11,000 × g for 10 min. The extracted mitochondria were used for the next study.

4 Reagent II (200 μL) and reagent III (2 μL) were added to the precipitate, and the precipitate was fully resuspended for the next step test of mitochondrial respiratory chain complex III and IV enzyme activity.

#### Detection of mitochondrial respiratory chain complex III enzyme activity

Reagent IV (25 μL) and working liquid (200 μL) were added to the microporous enzyme label plate, and after full mixing, the reaction time was accurate for 2 min. Then, after the 10 μL sample was added to fully mix, the initial absorption value A_1_ at 550 nm for 0 min and absorption value A_2_ after 2 min were immediately read, and ΔA = A_2_-A_1_ was calculated.

#### Detection of mitochondrial respiratory chain complex IV enzyme activity

The microporous enzyme label plate was successively added to 200 μL of working liquid and 10 μL of sample, and after full mixing, the initial light absorption values A_1_ at 550 nm for 0 min and A_2_ after 1 min were immediately read, and ΔA = A_1_-A_2_ was calculated.

### Metabolomics

Metabolome extraction were based on described methods with slight modifications (Hu et al., 2019). *Rhizoctonia solani* mycelia cakes were placed in 100 mL of potato glucose medium and oscillated in an incubator (ZQLY-1800 V, Shizhichu-electron, China) at 37°C and 180 rpm for 4 h, and then, antimycin A_1_ at a final concentration of 6.66 μg/mL was added to the broth. After continuous treatment for 24 h, the broth was centrifuged, and the precipitate was collected. Then, the mycelium was washed with 0.01 M sterile PBS 3 times to remove the medium. The mycelium was rapidly transferred to a centrifuge tube containing 1 mL of cold methanol (100%), rotated, mixed, and immersed in liquid nitrogen to quench the metabolites. Finally, the mycelium stored in liquid nitrogen was frozen and thawed three times to dissolve them, and then metabolites were centrifuged for 10 min at 10000 g at 4°C to collect the intracellular metabolites. Three biological replicates were performed in each group. Mycelia without antimycin A_1_ were used as the control group. Mycelia with antimycin A_1_ were used as the treatment group.

#### Liquid chromatography–mass spectrometry analysis

The sample was centrifuged at 4°C at 10000 × g for 3 min, and 200 μL of the supernatant was transferred to the corresponding inner tube of the sample vial for machine analysis. The supernatant was composed of a micro C_18_ column (2.1 mm × 100 mm, 1.8 μm) with a flow rate of 0.40 mL/min. The linear gradient of mobile phase a (ultrapure water, 0.1% formic acid) and mobile phase B (ACN, 0.1% FA) was 10–90%. The compounds were obtained in the Waters ACQUITY UPLC HSS T3 UHPLC system and detected by Triple TOF-6600 (AB Sciex) by tandem mass spectrometry.

#### Data processing

ProteoWizard software was used to convert the original data files obtained by LC–MS into mzML format. The peak extraction, peak alignment and retention time were calibrated by the XCMS program. The “SVR” method was used to correct the peak area, and the peaks with more than a 50% missing rate in each group were filtered. The filtered peaks were corrected. Compounds with accurate quality charge ratios (m/z) were labelled using METLIN,^1^ and other databases, including KEGG,^2^ genome.jp (kegg/), HMDB,^3^ and MetaboAnalyst^4^ (5.0 version), were used to identify metabolic pathways. Data were converted by LOG and processed in CTR format and analysed using SIMCA (version 16.0.2; Sartorius Stedim Data Analysis, UMEA, Sweden). The validity of the OPLS-DA model was evaluated with the total explanatory variance R^2^, predictive power Q^2^ and VIP score by repeated twofold cross-validation and replacement tests (*n* = 200). According to the importance of variables in the projection (VIP) value generated by OPLS-DA processing with a threshold of more than 1, the variables that contributed significantly to clustering and discrimination were identified. Student’s t test was used to detect differentially expressed metabolites, with a *p* value less than 0.05 and a fold-change (FC) greater than 2.0 or less than 0.5. Pathways with *p* < 0.05 were considered and filtered as the main metabolic pathways. In addition, heatmaps were constructed based on the relative levels of identified metabolites and plotted with R and gplot.

### RT–PCR analysis

Mycelia treated with antimycin A_1_ were washed with PBS 2 times and quickly transferred into a prepared mortar. Approximately 10 mL of liquid nitrogen was added, and the mycelia were fully ground into powder. The powder was transferred to a 1.5 mL RNA-free centrifuge tube and suspended in TRIzol to extract total RNA. Validamycin was used as a positive control. The RNA was then reverse-transcribed into cDNA. The mitochondrial genome of *Rhizoctonia solani* AG1 (MW995476.1) was searched from NCBI, a pair of gene-specific primers were designed using Primer Premier 5 on the upstream and downstream ORF of the genes to be detected (Table 1), and the chemical synthesis was commissioned by Shengong Bioengineering (Shanghai) Co., LTD. Using the c DNA of *R. solani* as a template, the Applied Biosystems StepOneTM Real-Time PCR System was used to detect the expression of ATP6, ATP8, COX1, COX2, COX3, ND1, ND3, ND4L, ND5, QCR6 and cytB in each mitochondria of *Rhizoctonia solani*. The fluorescent dye was ChamQ Universal SYBR qPCR Master Mix from Nanjing Nuoweizan Company.

**Table 1:** Inhibitory effects of antimycin A_1_ on *Rhizoctonia solani.*

Concentration (μg/mL)	Colony diameter (mm)			Average diameter (mm)	Inhibition ratio (%)
	1	2	3		
26.66	15.9	16.06	15.92	15.96 ± 0.05a	92.55
13.33	32.9	37.5	36.3	35.57 ± 1.38b	68.13
6.66	38.9	36.9	41.5	39.10 ± 1.33bc	63.63
3.33	43.1	41.9	40.8	41.93 ± 0.66 cd	60.13
1.66	48.8	43.7	47.9	46.80 ± 1.57de	54
0.83	48.9	47.6	53.9	50.13 ± 1.92e	49.88
0.416	66.4	52.5	65.7	61.53 ± 4.52f	35.63
CK	90	90	90	90.00 ± 0.00 g	0

CK is the control group. The results of Duncan’s multi-interval test showed that at the significance level of *p* < 0.05, different letters after ± SD indicated significant differences in the different concentration.

According to the protocol of RT–PCR kit (QIAGEN, Germany): The first step was predenaturation at 95°C for 1 min. The second step consisted of 10 s at 95°C, 15 s at 56 ~ 60°C, 30 s at72 °C, 40 cycles. In the third step, the dissolution curves of 95°C 15 s, 65°C 1 min, 95°C 15 s, 0.5°C/20 s were collected, and each sample was repeated three times. Ct value analysis and dissolution curve analysis were performed after the reaction. The method of $2^{-△△CT}$ was used to analyse the data, and the final result was made into a bar chart for comparison.

### Statistical analysis

All statistical analyses were carried out using GraphPad Prism 8.0.2 software. Using one-way analysis of variance (ANOVA) and Tukey’s test, *p* < 0.05 represented significant differences, and *p* < 0.01 was considered extremely significant. All data are expressed as the mean ± standard deviation (SD).

## Results

### Inhibitory effect of antimycin A_1_ on hyphal growth of *Rhizoctonia solani*

It can be clearly seen from Table 1 that the growth of *Rhizoctonia solani* standing on the plate supplemented with antimycin A_1_ was inhibited compared with the blank control group. With the increase in the concentration of antimycin A_1_, the antifungal activity of *Rhizoctonia solani* also increased continuously, and the colony diameter decreased. When the concentration of antimycin A_1_ was 26.66 μg/mL, the bacteriostatic rate reached 92.55%. The EC_50_ value was 1.25 μg/mL. The colony diameter of the hyphal body in the antimycin A_1_ treatment group did not show a linear relationship with the antimycin A_1_ concentration. When the concentration of antimycin A_1_ was less than 5 μg/mL, the change in antifungal activity increased with increasing concentration of antimycin A_1_, and when the concentration was greater than 5 μg/mL, the change in antibacterial activity did not increase with increasing concentration of antimycin A_1_. It could be inferred that when the concentration of antimycin A_1_ was relatively small, it played an inhibitory role, and when the concentration was certain, antimycin A_1_ could kill *Rhizoctonia solani*.

### Dehydrogenase activity measurement

**Figure 1:**
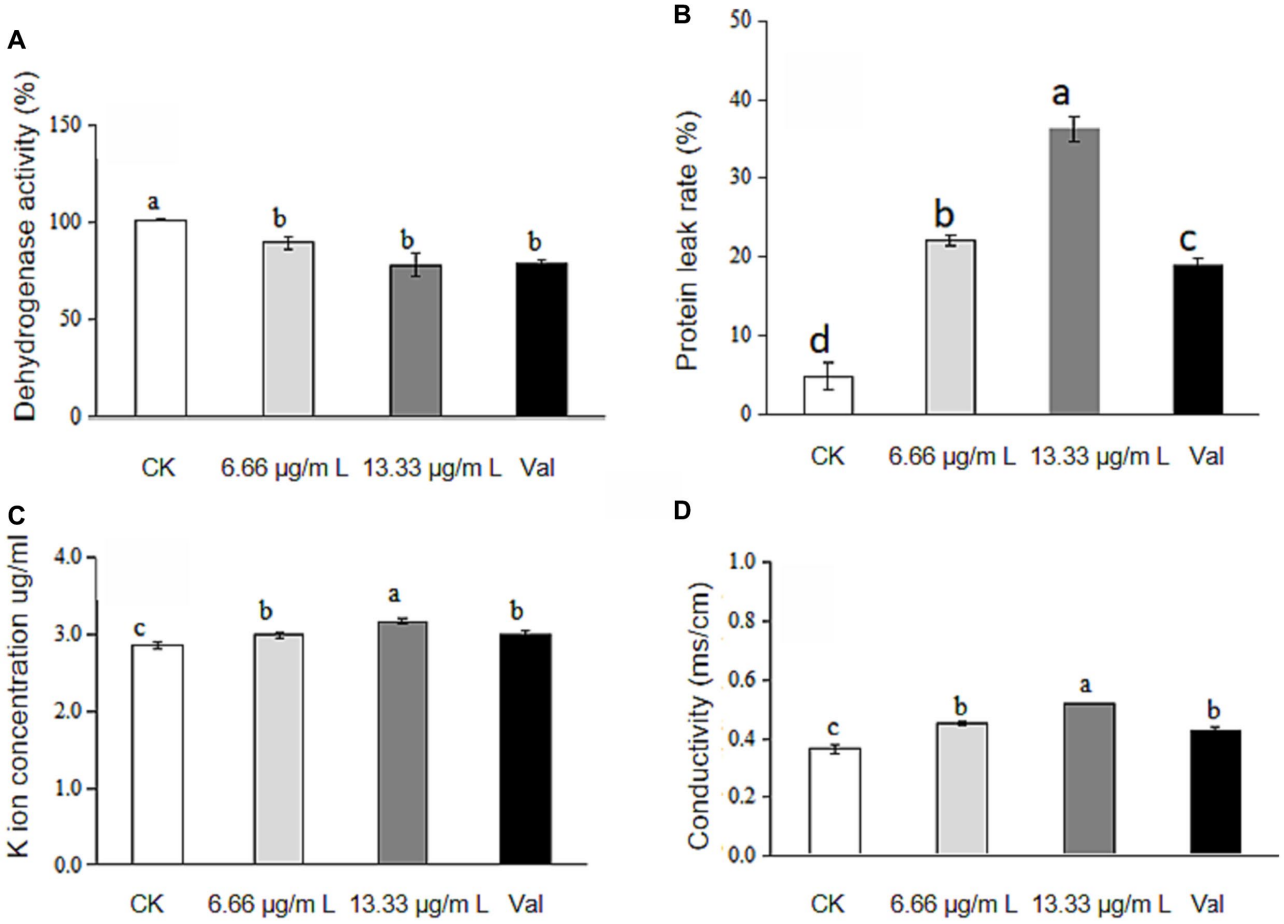
Changes in dehydrogenase activity, leakage of protein and K ions and conductivity of Rhizoctonia solani culture medium treated with antimycin A1. **(A)** Changes in dehydrogenase activity; **(B)** Leakage of protein. **(C)** Leakage of K ions; **(D)** Conductivity. Note. CK indicates the control group. Val indicates validamycin. Values are mean ± S.E. and different letters means significant difference (*n* = 3, *p*<0.05).

The absorbance of each gradient sample was measured at a wavelength of 485 nm, and 3 parallel samples were set for each group. According to the principle of the TTC method, the change in intracellular dehydrogenase activity was obtained according to the calculation formula of dehydrogenase activity. The data indicate a reduction in dehydrogenase activity to 81.96 and 77.96% at concentrations of 6.66 μg/mL and 13.33 μg/mL, respectively, following the treatment with antimycin A_1_, as depicted in Figure 1A. For different concentrations of antimycin A_1_, the higher the concentration of antimycin A_1_ solution was, the more the dehydrogenase activity of mycelia decreased, indicating that antimycin A_1_ reduced the dehydrogenase activity of mycelia.

### Protein leakage

After mycelia of *Rhizoctonia solani* were treated with different concentrations of antimycin A_1_ for 2 h, the absorbance of each gradient sample was measured at 595 nm wavelength, and 3 parallel samples were set for each group. According to the calculation formula of protein leakage rate, the change of protein leakage rate in *Rhizoctonia solani* cells was obtained. Compared with the blank control group, the release of protein in mycelial cells was significantly increased. Results shown that protein release in mycelial cells increased by 17.3 and 31.4%, respectively after treatment with 6.66 μg/mL and 13.33 μg/mL antimycin A_1_ in Figure 1B. The higher the concentration of antimycin A_1_, the higher the protein leakage rate of *Rhizoctonia solani* cells. Antimycin A_1_ could damage the integrity of the cell membrane of mycelia and release proteins in the cells. It could be seen that antimycin A_1_ could promote the protein leakage in *Rhizoctonia solani* cells.

### K^+^ leakage was detected in the culture medium of *Rhizoctonia solani*

The absorbance of each gradient sample was measured by an atomic absorption spectrophotometer, 3 parallel samples were set for each group, and the measured values were summarized and analysed. As shown in Figure 1C, compared with the blank control group, after treatment with antimycin A_1_, the potassium (K^+^) leakage rate from Rhizoctonia solani was found to elevate to 4.77% at a concentration of 6.66 μg/mL and to 11.08% at 13.33 μg/mL. For different concentrations of antimycin A_1_, the higher the concentration of antimycin A_1_ was, the higher the K^+^ leakage rate of *Rhizoctonia solani* cells, indicating that antimycin A_1_ promoted K^+^ leakage in the cells of *Rhizoctonia solani*.

### The change in electrical conductivity in the culture medium of *Rhizoctonia solani*

Changes in membrane permeability could be reflected by detecting changes in electrical conductivity. Figure 1D illustrated that the conductivity of the mycelium culture medium increased following treatment with antimycin A1, reaching 24.47% at a concentration of 6.66 μg/mL and 43.01% at 13.33 μg/mL across the treated groups, indicating that antimycin A_1_ could cause the leakage of substances in the mycelium cells of *Rhizoctonia solani*, and the effect of increasing the conductivity of mycelium culture medium became increasingly obvious as the concentration of antimycin A_1_ increased. These results indicated that antimycin A_1_ could destroy the cell membrane of mycelia and induce electrolyte extravasation in the cells of *Rhizoctonia solani.*

### Influence of antimycin A_1_ on the activity of electron transport chain complex III and IV of *Rhizoctonia solani*.

The activity of electron transfer chain complexes III and IV decreased, and the concentration of antimycin A_1_ was negatively correlated with the activity of electron transfer chain complexes III and IV. At a concentration of 6.66 μg/mL, the activity of electron transport chain complexes III and IV was 55 and 88% that of the control group, respectively. At 13.33 μg/mL, the activity of electron transport chain complexes III and IV was 51 and 55% that of the control group, respectively (Tables 2, 3).

**Table 2:** Effects of antimycin A_1_ on the activity of electron transport chain complex III (coenzyme Q-cytochrome C reductase) of *Rhizoctonia solani*.

Concentration (μg/mL)	Activity of enzyme(U/min/mg prot)			
	1	2	3	Mean ± SD
CK	20.75	14.9	12.18	15.77 ± 2.57a
6.66	9.77	10.69	5.75	8.74 ± 1.51ab
13.33	9.69	10.01	4.51	8.07 ± 1.78b
Val	2.81	8.80	11.62	7.74 ± 2.60b

CK is the control group. The results of Duncan’s multi-interval test showed that at the significance level of *p* < 0.05, different letters after ± SD indicated significant differences in the scores of the same period.

**Table 3:** Effects of anti-amycin A_1_ on the activity of electron transport chain complex IV (cytochrome c oxidase) in *Rhizoctonia solani*.

Concentration (μg/mL)	Activity of enzyme(U/min/mg prot)			
	1	2	3	Mean ± SD
CK	20.75	14.9	12.18	15.77 ± 2.57a
6.66	9.77	10.69	5.75	8.74 ± 1.51ab
13.33	9.69	10.01	4.51	8.07 ± 1.78b
Val	2.81	8.80	11.62	7.74 ± 2.60b

CK is the control group. The results of Duncan’s multi-interval test showed that at the significance level of *p* < 0.05, different letters after ± SD indicated significant differences in the scores of the same period.

### The effects of antimycin A_1_ on the hyphal morphology of *Rhizoctonia solani*

**Figure 2:**
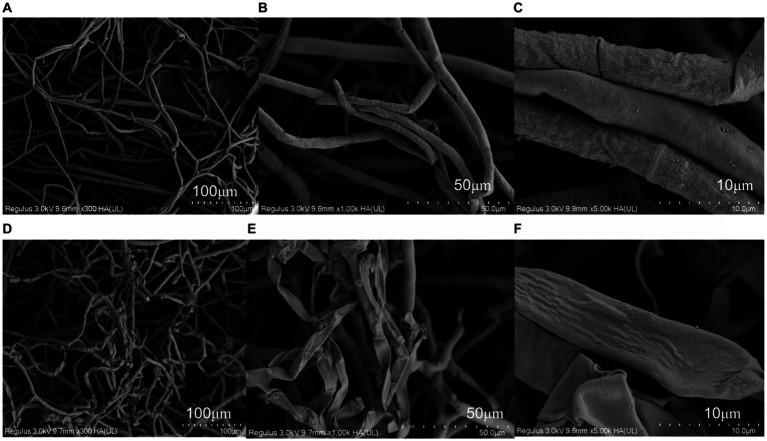
Effect of anti-amycin A_1_ on the mycelial morphology of Rhizoctonia solani under a scanning electron microscope. **(A–C)** Scanning electron microscope image of Rhizoctonia solani mycelia in the control group. **(D–F)** Scanning electron microscopy image of Rhizoctonia solani mycelia treated with antimycin A_1_. **(A,D)** magnification ×300, **(B,E)** magnification ×1,000, **(C,F)** magnification × 5,000.

The results of scanning electron microscopy (SEM) are shown in Figure 2. Mycelia in the control group were full in shape, smooth in surface, sparse in distribution, ends were complete and semicircular without fracture, and long branches could be formed. After treatment with antimycin A_1_, the mycelium morphology was shriped, and the surface was rough and folded. The mycelia were seriously distorted and broken, ends were incomplete or locally enlarged, branches were short, and the number was reduced. The above results indicated that antimycin A_1_ had a serious destructive effect on the morphology and structure of the hyphae of *R. solani*.

**Figure 3:**
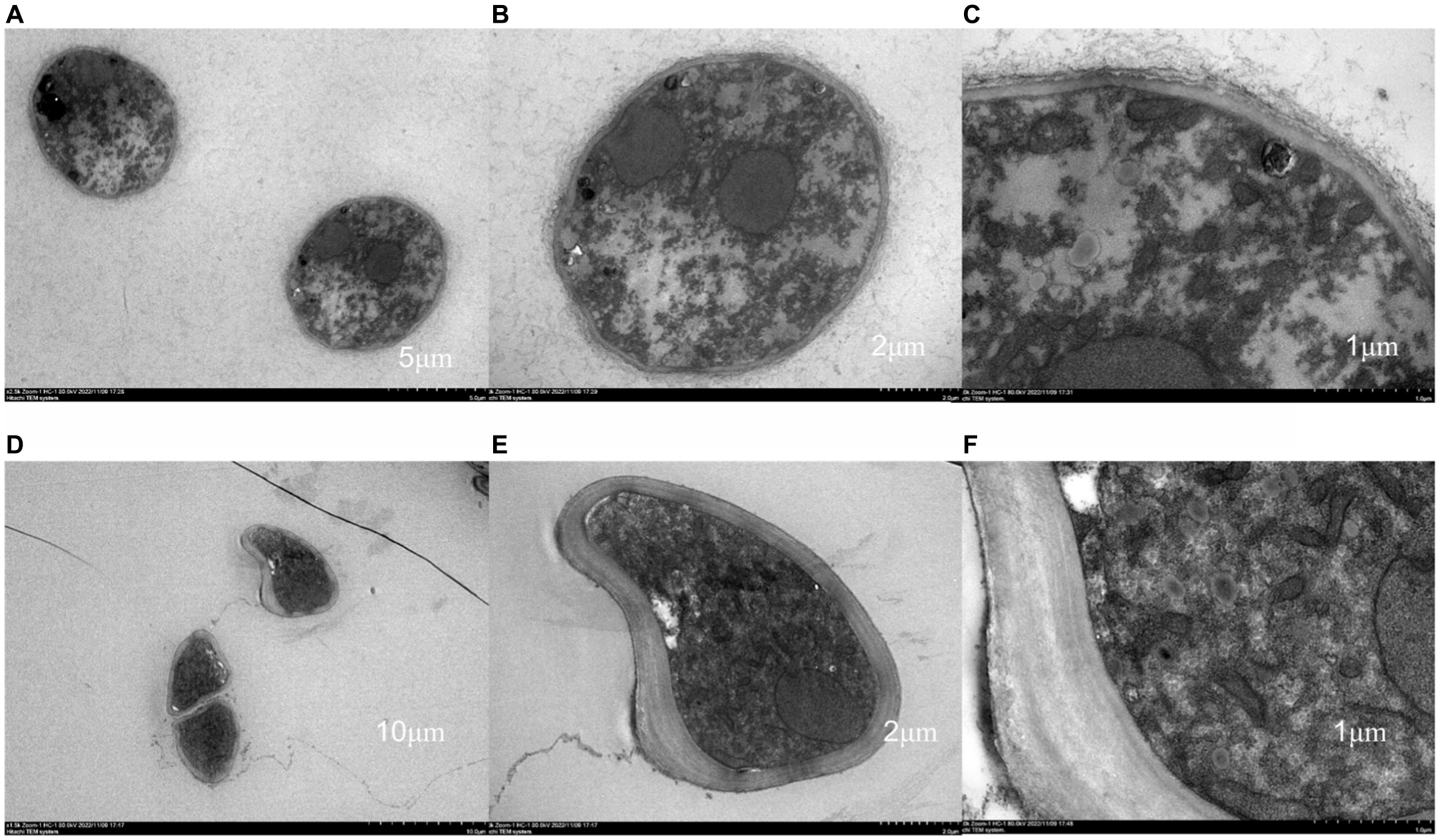
Effect of antimycin A_1_ on the mycelial morphology of Rhizoctonia solani under a transmission electron microscope. **(A–C)** Transmission electron microscopy of Rhizoctonia solani mycelia in the control group. **(D–F)** Transmission electron microscopy image of Rhizoctonia solani mycelia treated with antimycin A_1_. **(A,D)** magnification ×2,500, **(B,E)** magnification ×5,000, **(C,F)** magnification × 15,000.

The results of transmission electron microscopy (TEM) are shown in Figure 3. In the control group, the materials inside the mycelium were evenly distributed, and the cell wall, membrane, mitochondria and vesicles were normal and evenly distributed, with a large number of mitochondria, and the cell wall and membrane were intact. After treatment with antimycin A_1_, the cell morphology changed significantly, the cell wall became thick, the organelle boundary was not obvious, mitochondria were swollen, substances in vesicles were exosmotic, vesicles slowly fused and cavitation occurred. The above results indicated that the morphology and structure of mycelia were significantly changed after treatment with antimycin A_1_, especially changes in mitochondria. Therefore, the effect of antimycin A_1_ on mycelia was closely related to mitochondria.

### Metabolomics analysis

**Figure 4:**
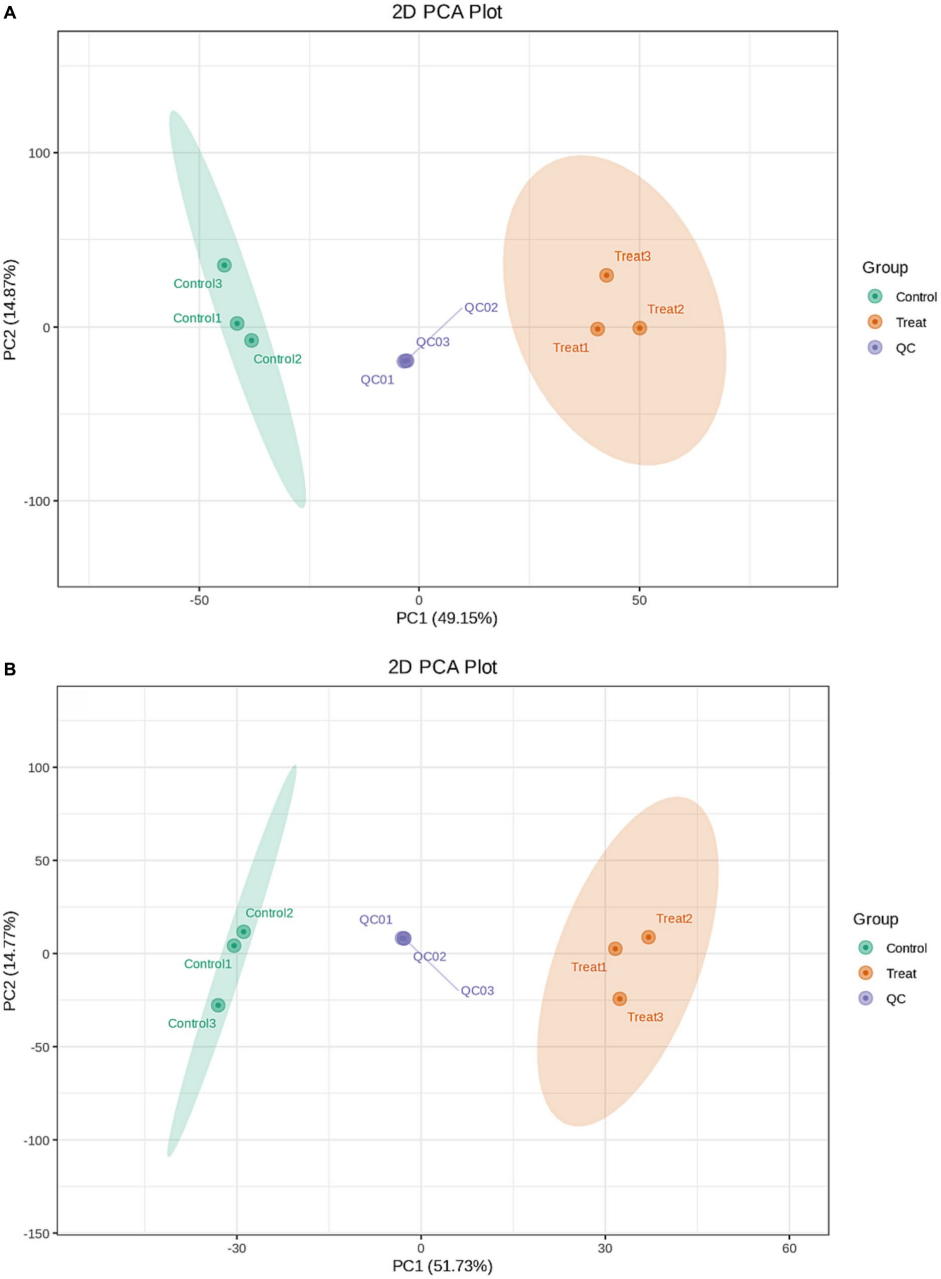
PCA score plots for the mass spectrum data of the control group, treatment group, and quality control sample. **(A)** Positive mode; **(B)** Negative mode.

During instrumental analysis, one quality control sample was inserted into every three test analysis samples to monitor the repeatability of the analysis process. A total of 4,002 metabolites were detected. A total of 2,640 metabolites were detected in the cationic model, and 1,362 metabolites were detected in the anionic model. The metabolic changes between the control group and the treatment group were analysed by principal component analysis. The positive ion mode and the negative ion mode are shown in Figure 4. The X-axis represented the first principal component (PC1), which accounted for 49.15% (the positive ion mode) and 51.73% (the negative ion mode) of the data variability. The Y-axis represented the second principal component (PC2), which accounted for 14.87% (the positive ion mode) and14.77% (the negative ion mode) of the data variability. Together, these two principal components explained a total of 64.02% (the positive ion mode) and 66.5% (the negative ion mode) of the variability and showcased the overall structure of the data captured by the two components. The plot reveals how three different sample groups are distributed within the space formed by PC1 and PC2. The control group (Control) is primarily located on the left side of the plot, the treatment group (Treat) is mainly concentrated on the right side, and the quality control group (QC) is distributed around the center. This suggested that there were significant differences in the sample characteristics between the control group and the treatment group, while the positioning of the quality control group might indicate its role as a stable reference point for the experiment. Orthogonal partial least squares discriminant analysis (OPLS-DA) was used for metabolomic analysis. The metabolic profiles of the treatment group and the control group were obviously separated. The interpretation rate and prediction rate of the OPLS-DA model were R^2^Y = 1 and Q^2^ = 0.96, respectively. The results show that the OPLS-DA model was reliable without overfitting; this also suggested that antimycin A_1_ affected the metabolic process of *Rhizoctonia solani*, leading to changes in its metabolites.

**Figure 5:**
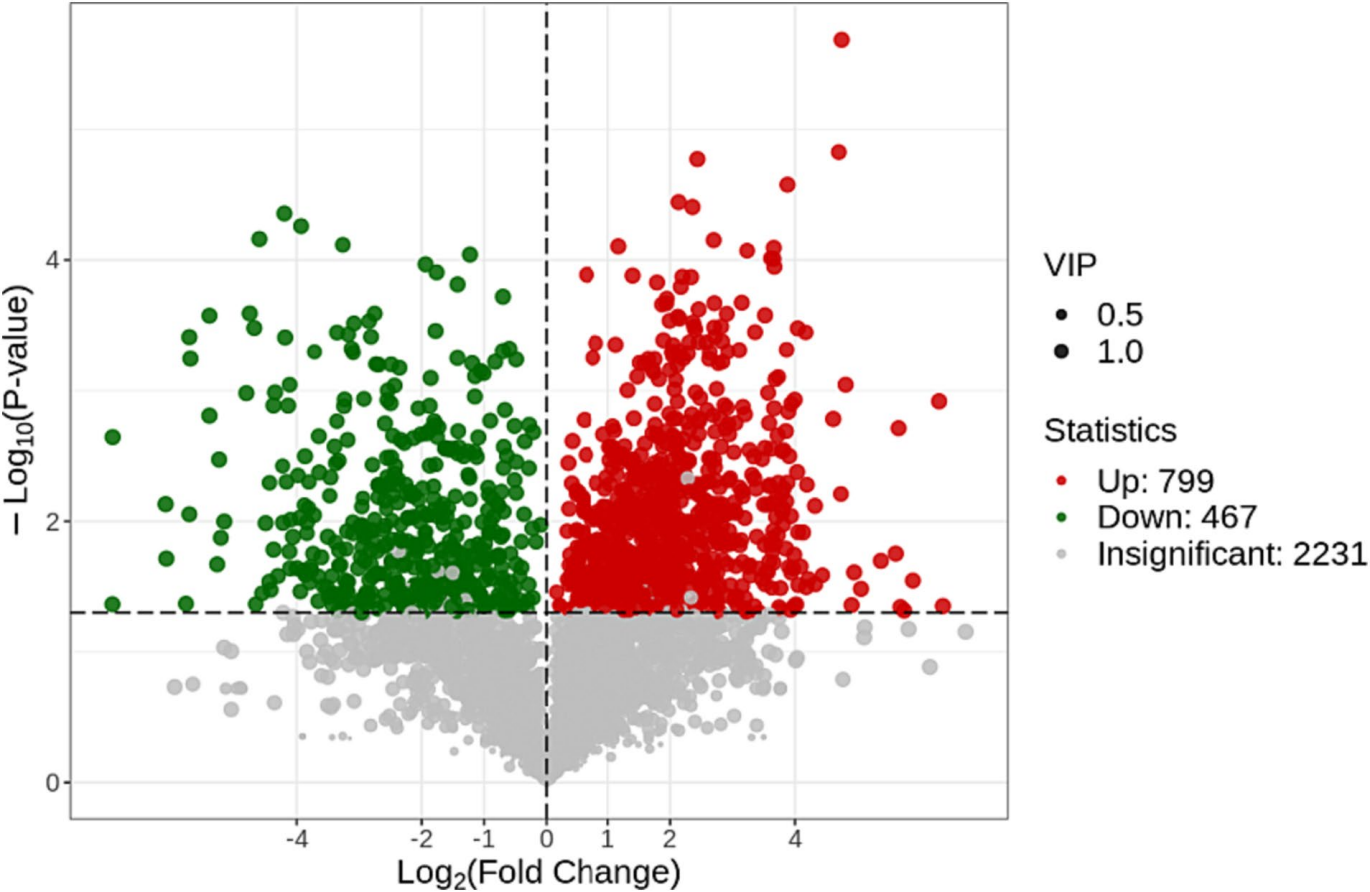
Volcano plot for differential metabolites of the control group and the treatment group.

The volcano map was used to show the difference and significance of the differences between the two groups of samples. In this plot: the X-axis represents the logarithm of the fold change (Log2 (Fold Change)), indicating the degree of upregulation or downregulation of metabolites. Positive values indicate upregulation (increase) in metabolites, while negative values indicate downregulation (decrease) in metabolites. The Y-axis represents the negative logarithm of the value of p (−Log10 (*p*-value)), representing statistical significance. The higher the value, the higher the statistical significance. This category included 467 downregulated metabolites (green dots) and 799 upregulated metabolites (red dots), with grey representing metabolites detected with no significant difference (Figure 5).

**Figure 6:**
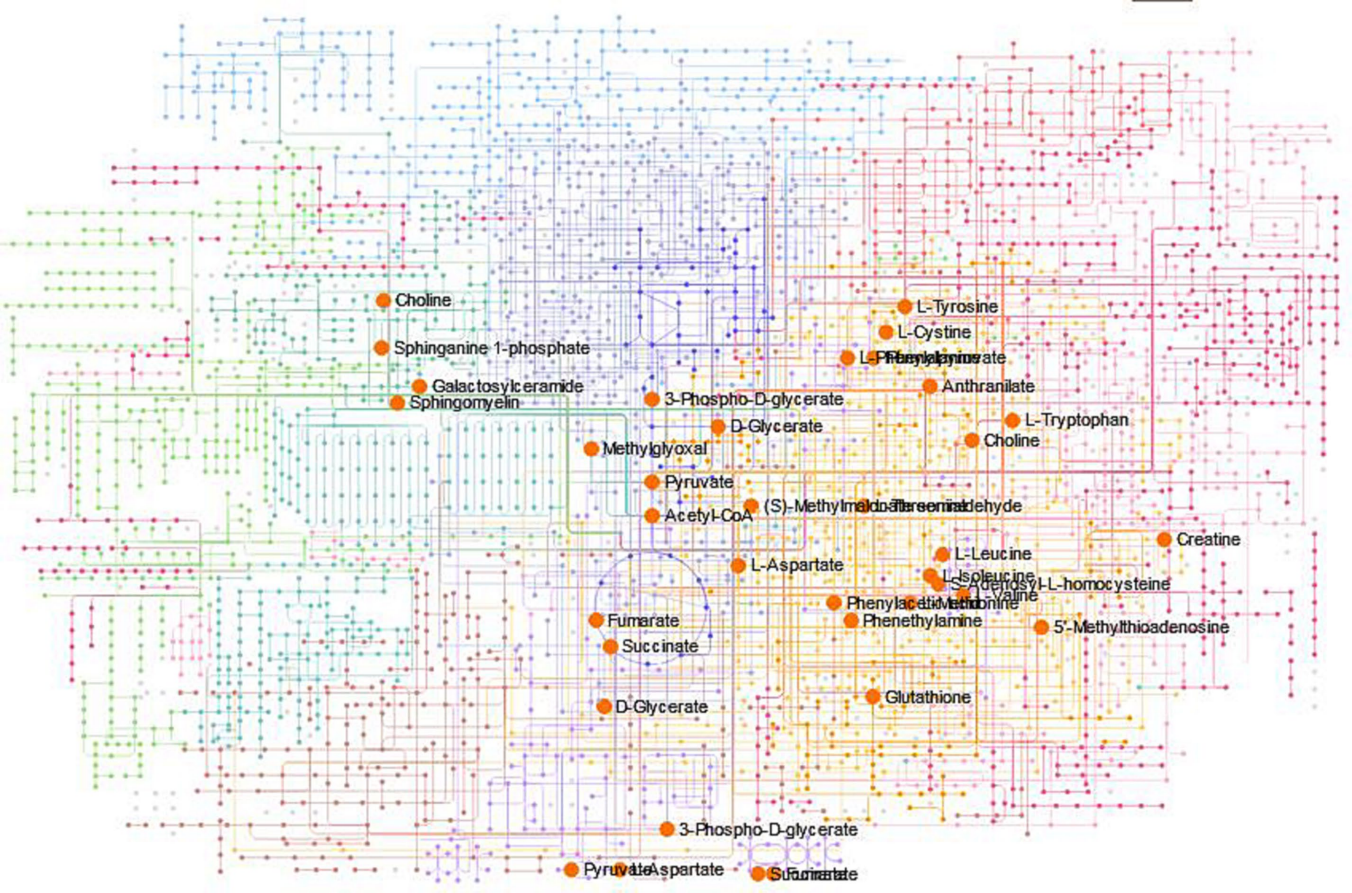
The KEGG global metabolic network.

To determine intermediate metabolites that played a direct role in the antimicrobial process, we found the major metabolic pathways. According to KEGG database analysis, 12 metabolic pathways had significant differences (*p* < 0.05, Impact>0): phenylalanine, tyrosine and tryptophan biosynthesis, valine, leucine and isoleucine biosynthesis, cysteine and methionine metabolism, glycine, serine and threonine metabolism, phenylalanine metabolism, valine, leucine and isoleucine degradation, citrate cycle (TCA cycle), alanine, aspartate and glutamate metabolism, pyruvate metabolism, sulfur metabolism, glyoxylate and dicarboxylate metabolism and propanoate metabolism (Supplementary Figure S1; Supplementary Table S2). This image (Supplementary Figure S2) showed simplified diagrams of two metabolic pathways, with arrows and compound codes indicating the individual steps within the pathways. The red squares represented metabolites that had significantly changed in quantity under experimental conditions, while the blue squares indicated metabolites that had not shown significant changes or were not covered in the study. By employing the metabolite codes from the KEGG database, one could identify and look up these specific compounds and their roles in biological pathways. These also involved 49 metabolites, such as 3-dehydroshikimate, prephenate, pyruvic acid, among others (Tab S3). The Network Explorer analysis module enhanced the joint pathway analysis module of MetaboAnalyst by enabling the detection of connections that extend across pathway boundaries. This feature expanded our understanding of the pathways, providing a more comprehensive view. Figure 6 illustrated the KEGG global metabolic network. The image was a complex network map that displayed a multitude of intersecting and overlapping pathways, indicating a comprehensive overview of metabolic or biochemical pathways. Colored lines represented individual biochemical pathways, indicating the flow and interconnection of various metabolic processes. Circles (nodes) containing the names of compounds signified key metabolites within these pathways. Some compounds such as (S)-methylmalonic acid semialdehyde, pyruvic acid, choline, fumaric acid, succinic acid, among others were displayed in a larger size and marked in orange, possibly denoting their higher importance or relevance within this particular network.

### RT–PCR

Using ATP9 as an internal reference, the data obtained by RT–PCR were analysed by the comparative $C_{T}$ value method ( $2^{-△△CT}$ method), and a histogram of gene expression was obtained. The results showed that the expression of seven genes, ATP6, ATP8, COX3, QCR6, CytB, ND1 and ND3, in the mitochondria of *Rhizoctonia solani* treated with antimycin A1 was downregulated, and the results of variance analysis showed significant differences in the expression levels of these seven genes (Supplementary Figure S3). The data in the graph represents the average of 10–12 replicates ± standard error. The Duncan’s new complex range method was employed for significant difference analysis. Different letters above the columns indicate significant differences (*p* < 0.05). There was a statistically significant difference between the antimycin A_1_ treatment group and the blank control group. There was also a statistically significant difference between the positive control group and the blank control group (*p* < 0.05). Compared with the blank control group, there was a significant difference between the antimycin A_1_ treatment group and the positive control group (p < 0.05). There was no significant difference in the gene expression levels of COX1. The gene expression levels of ND5, ND4L and COX2 in the three genes treated with antimycin A_1_ decreased and had significant differences compared with the blank control group, but the gene expression levels of the positive control group were all increased compared with the blank control group.

## Discussion

### The inhibitory effect of antimycin A_1_ against *Rhizoctonia solani*

At present, more than 40 naturally derived antimycin compounds have been discovered, most of which have broad-spectrum antibacterial effects. For example, Shiomi found that antimycin A_9_ had a variety of antibacterial activities, and the resistance effect against *Trichophyton mentagrophytes* was most significant (Shiomi et al., 2005). Antimycin A_10_-A_16_ and antimycin A_3_ also showed significant resistance to *Candida utilis* (Hosotani et al., 2005). Recent studies have found that at 10 μg/mL, antimycin A inhibited mycelial growth (62.90%), conidiogenesis (100%), and conidia germination (42%) of *Magnaporthe oryzae Triticum* (Paul et al., 2022). In addition, some antimycin analogues have been found to have antifungal activity (Yan et al., 2013). Antimycin A_1_ also exhibited similar antifungal activity against multiple fungi. The results showed that the inhibitory rate of antimycin A_1_ reached 92.55% when the concentration of antimycin A_1_ was 26.66 μg/mL. The EC_50_ value was 1.25 μg/mL.

### Influence of antimycin A_1_ on the morphology and ultrastructure of mycelia of *Rhizoctonia solani*

The hyphal morphology of *R. solani* treated with antimycin A_1_ at a concentration of 6.66 μg/mL was locally twisted and partly shrinking, and the surface was rough and folded. Differences existed between antimycin A_1_ and the other fungicide used to control *R. solani.* Validamycin caused abnormal branching at the tips of hyphae and inhibited their continuous growth (Iwasa et al., 1971). DPZ-8 was a metabolite secreted by *Streptomyces padanus* JAU4234 that leads to instantaneous shortening of the hyphal nodes and contraction of the hyphae (Wei et al., 2012). There were other possible causes for the different characteristics of these pesticides. The above results indicated that antimycin A_1_ had a serious destructive effect on the morphology and structure of the hyphae of *R. solani*. After treatment with antimycin A_1_, the cell ultrastructure of mycelia of *R. solani* changed significantly. The mycelial cell wall became thick, the organelle boundary was not obvious, the mitochondria were swollen, the substances in the vesicles were exosmotic, and the vesicles slowly fused and cavitation occurred. The TTC method was used to measure dehydrogenase activity. The results showed that dehydrogenase activity decreased with increasing antimycin A_1_ concentration. According to the determination experiment of the protein and K ion leakage rates, as the concentration of antimycin A_1_ increased, the leakage rates of both proteins and K ions increased. Therefore, it could be inferred that antimycin A_1_ should destroy the cell membrane structure and lead to the leakage of intracellular substances, which was consistent with the phenomenon observed under the electron microscope. The effect of antimycin A_1_ on mycelia was closely related to mitochondria. Electron transport chain complexes III and IV are necessary for electron transport in the reaction and directly affect continuous electron transport (Antal et al., 2013). Antimycin A_1_ significantly increased the production of reactive oxygen species and caused ATP inhibition and glutathione depletion (Park and You, 2016). The production of reactive oxygen species (ROS) and the depolarization of the membrane mediated by antimycin A_1_ lead to apoptosis, thus releasing pro-apoptotic molecules such as cytochrome c into the cytoplasm (King and Radicchi-Mastroianni, 2002; King, 2005). Although the activity of antimycin A_1_ in mammalian cells has been reported by researchers, there have been few reports on antimycin A_1_ controlling the antifungal activity of plant pathogenic fungi (Paul et al., 2022). To verify the inhibitory effect of antimycin A_1_ on the electron transport chain of fungi, we conducted experiments using electron transport chain complexes III and IV. It was found that antimycin A_1_ could significantly inhibit mitochondrial complex III and complex IV of *R. solani*, which was similar to previous results (Cai and Jones, 1998). In order to validate the potential damaging effect of antimycin A_1_ on fungal mitochondria, a selection of mitochondrial genes was chosen for experiments. The RT-PCR test results revealed that the expression of seven genes, ATP6, ATP8, COX3, QCR6, CytB, ND1, and ND3, in the mitochondria of *R. solani* treated with antimycin A_1_ was significantly downregulated. This further confirms the inhibitory effect of antimycin A_1_ on the fungal mitochondrial respiratory chain.

### Pathway topology analysis

The Pathway Analysis module synergistically integrates results from robust pathway enrichment analysis with pathway topology analysis to identify the most relevant pathways affected by the administration of antimycin A_1_. To determine intermediate metabolites that played a direct role in the antimicrobial process, we found the major metabolic pathways. According to pathway topology analysis, 12 metabolic pathways had significant differences (*p* < 0.05, Impact>0), including phenylalanine, tyrosine and tryptophan biosynthesis. This also involved 12 metabolites, such as 3-dehydroshikimate, among others. The biosynthesis of tryptophan, tyrosine, and phenylalanine occurred through a common pathway of chorismate, at which point the pathway branches, one branch proceeded to tryptophan, and the other to tyrosine and phenylalanine (Xie et al., 2000; Ferrer et al., 2022). This pathway is an attractive target of nontoxic fungicides (Gurvan et al., 2003). Compared with the control group, the above 12 metabolites in the treatment group were significantly upregulated. The results showed significant changes in phenylalanine, tyrosine and tryptophan biosynthesis metabolic pathways in the treatment group compared to the control group.

Pathway topology analysis revealed that the biosynthesis pathways of valine, leucine, and isoleucine were notably significant, involving key metabolites such as acetyl-CoA and pyruvic acid, among others. Beyond these two pathways, the analysis also highlighted the substantial significance of cysteine and methionine metabolism pathways. These pathways include a different set of critical metabolites, namely acetyl-CoA, (S)-methylmalonic acid semialdehyde, succinic acid, and fumaric acid, among others.

Pyruvic acid is an important intermediate that participates in the metabolism of carbohydrates, proteins, and fats. Pyruvate serves as a biological fuel by being converted to acetyl coenzyme A, which enters the tricarboxylic acid or Krebs cycle where it is metabolized to produce ATP aerobically. Energy could also be obtained anaerobically from pyruvate via its conversion to lactate. The results showed significant changes in metabolic pathways in the treatment group compared to the control group. These pathways likely experienced alterations in their metabolite profiles due to the inhibitory effects of antimycin A_1_.

The current research into the efficacy of antimycin A_1_ against specific pathogenic bacteria has certain limitations: First, the scope of testing was confined to *Rhizoctonia solani*, which limits the generalizability of our findings to other fungal pathogens. Sensitivity and resistance to antimycin A_1_ can vary significantly among different pathogens, and this variability has not been explored in this study. Second, the bioavailability of antimycin A_1_ within plants, which is a critical determinant of its effectiveness when applied to crops, was not investigated in this study. As such, further research is required to ascertain the bioavailability of antimycin A_1_ and its real-world effectiveness in agricultural settings.

## Conclusion

The effects of antimycin A_1_ on the mycelia of *R. solani* were primarily characterized by abnormal mycelia, including local distortion, bifurcation, enlargement of the cavity, and malformation of the cell wall. The mechanism of action of antimycin A_1_ involved targeting the mitochondria. It was discovered that antimycin A_1_ inhibited the activity of mitochondrial electron transport chain complexes III and IV, thereby suppressing the respiration of *Rhizoctonia solani*. A metabolomic analysis revealed that antimycin A_1_ significantly impacted 12 metabolic pathways. Due to the inhibitory effects of antimycin A_1_, it is highly likely that these pathways underwent changes in their metabolite profiles. These findings have significant implications for the potential development of novel fungicides. By understanding the specific effects of antimycin A_1_ on the mycelia of *R. solani*, researchers can explore its potential as a fungicide and develop more effective strategies for controlling fungal pathogens.

## Data availability statement

The original contributions presented in the study are included in the article/Supplementary material, further inquiries can be directed to the corresponding author.

## Ethics statement

The manuscript presents research on animals that do not require ethical approval for their study.

## Author contributions

LZ: Formal Analysis, Investigation, Methodology, Writing – original draft. CW: Investigation, Writing – original draft, Data curation. XS: Investigation, Visualization, Writing – review & editing. XZ: Funding acquisition, Project administration, Resources, Writing – review & editing.

## Footnotes

^1^ http://metlin.scripps.edu/

^2^ http://www.kegg

^3^ http://www.hmdb.ca/

^4^ https://www.metaboanalyst.ca/
