# Supplementary material for: Aptly chosen, effectively emphasizing the action and mechanism of antimycin A1
Source: Front Microbiol. 2024 Apr 3;15:1371850. doi: 10.3389/fmicb.2024.1371850 (PMC11021728; doi:10.3389/fmicb.2024.1371850)
Supplement: Supplementary file 1 [file Data_Sheet_1.PDF]

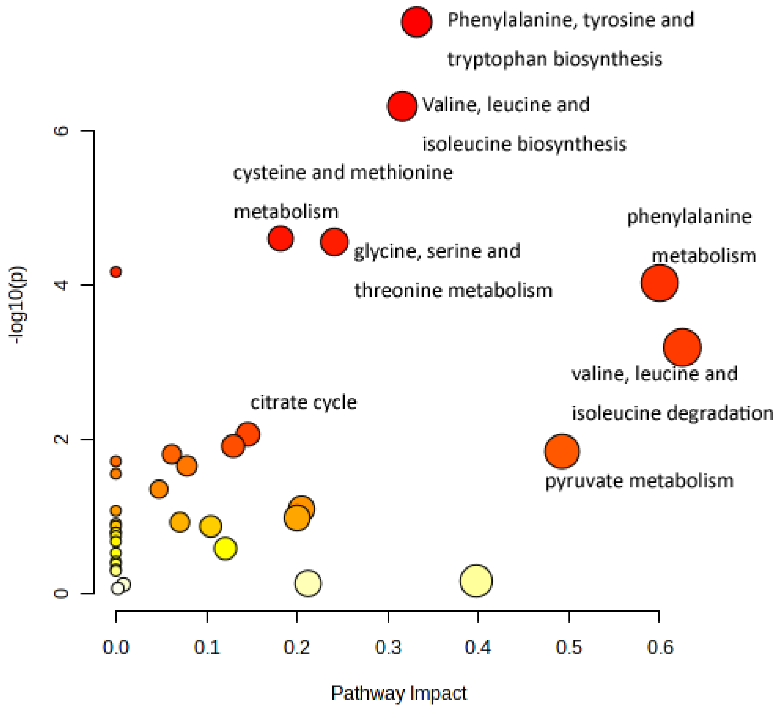

FIGURE S1 The major metabolic pathways of the control group and the treatment group

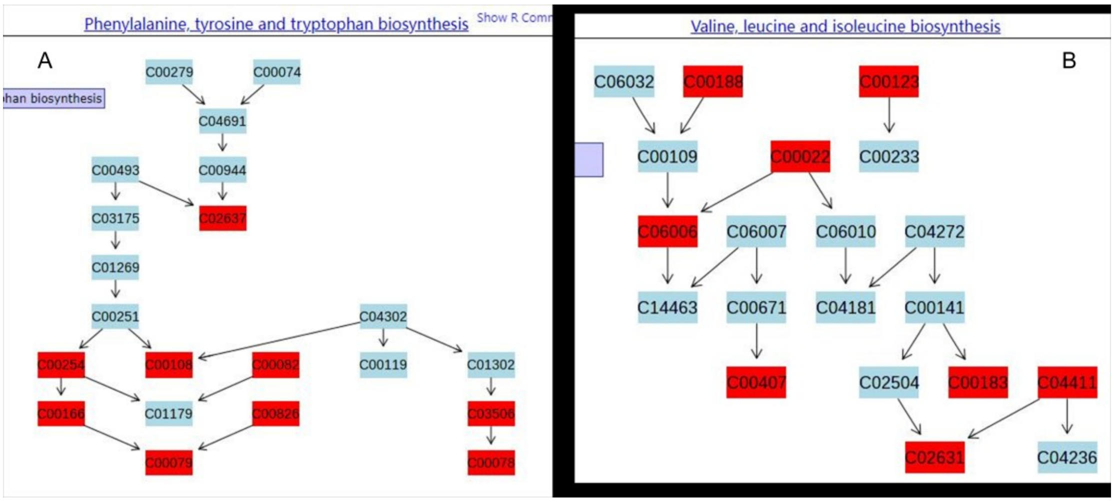

FIGURE S2 The metabolic pathways of A, phenylalanine, tyrosine and tryptophan biosynthesis and B, valine, leucine and isoleucine biosynthesis
