# Supplementary material for: Aptly chosen, effectively emphasizing the action and mechanism of antimycin A1
Source: Front Microbiol. 2024 Apr 3;15:1371850. doi: 10.3389/fmicb.2024.1371850 (PMC11021728; doi:10.3389/fmicb.2024.1371850)
Supplement: Supplementary file 2 [file Data_Sheet_2.PDF]

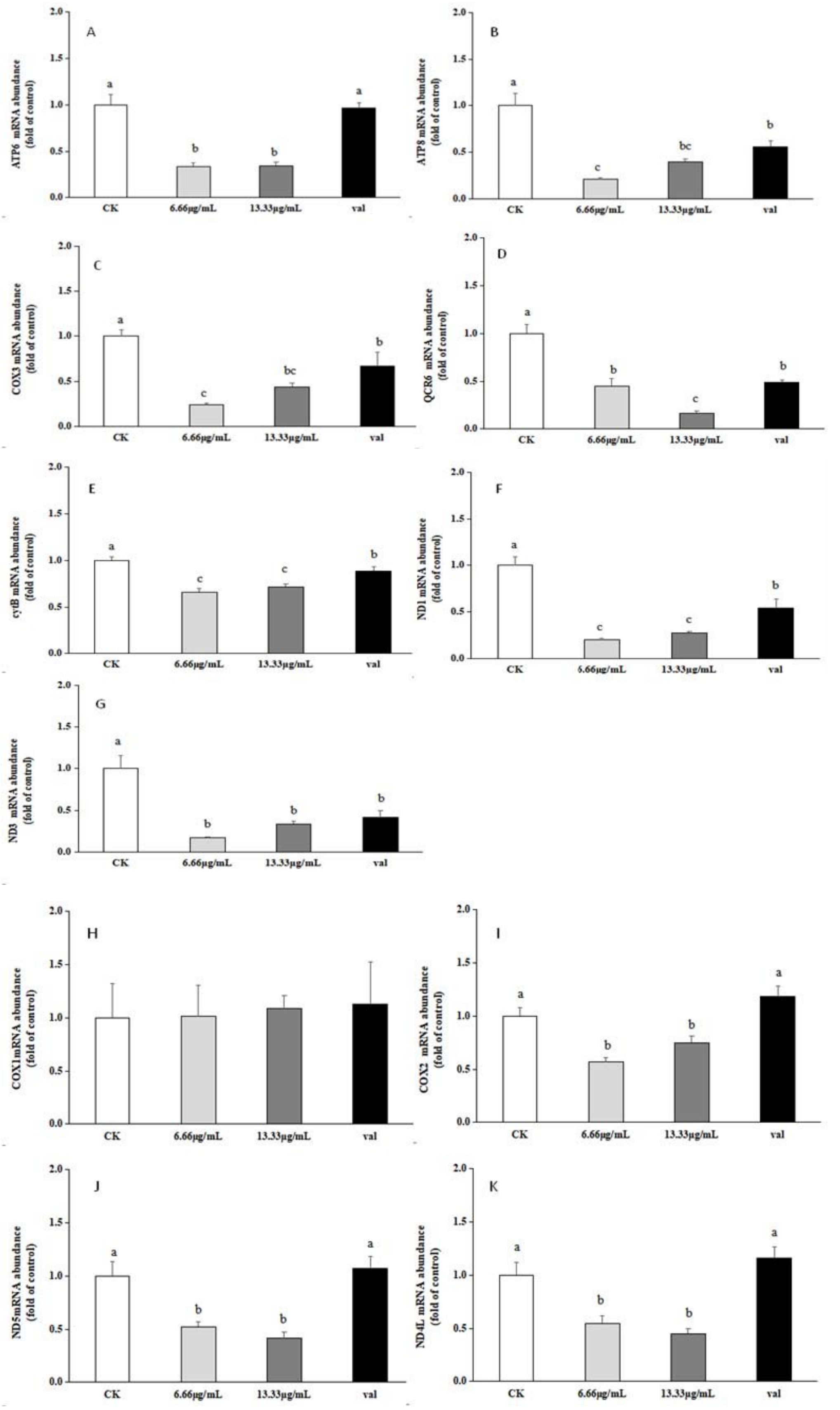

FIGURE S3 Expression changes of genes of *Rhizoctonia solani* treated with antimycin A_1

Note: Using ATP9 as the internal reference, the relative gene expression was measured by fluorescence quantitative PCR. The data in the figure are the mean ± standard error of three replicates. Duncan's new complex range method (DMRT) was used for significant difference analysis.
