# Supplementary material for: Aptly chosen, effectively emphasizing the action and mechanism of antimycin A1
Source: Front Microbiol. 2024 Apr 3;15:1371850. doi: 10.3389/fmicb.2024.1371850 (PMC11021728; doi:10.3389/fmicb.2024.1371850)
Supplement: Supplementary file 3 [file Data_Sheet_3.PDF]

TABLE S1 Primers for PCR amplification

Genes	Forward Primer (5’-3’)	Reverseprimer(5’-3’)
ATP6	TAGGAACAGCGAATGAAGTA	GGGATAAAGAATGAGAAGAA
ATP8	TGCATTAATATATGTGTTTT	AGATTTGTTGCTTAGTTTTG
COX1	GTGAACTATCTTGTGCCT	TAACCTGTCCATCCTGTA
COX2	TAAAGTAGCAGGGCACCAAT	CGACCAGGAACAGCATCAAT
COX3	TCTTTCAAGCGGTGCTACTG	ATGATGCTCGTCTATTTCCT
ND1	AGAGCAACATTACCTCGATTAAGA	CTATAAGCAGTCCCGGTACTAAAC
ND3	ATTTATATTTGTTCCTGTTT	GTATGCTTGTACTTTTTCTG
ND4L	TTAGTTCTTTTGGTTTCGAT	GACTCTGCTCCTGCTATTGC
ND5	TATGTTTATCGGTTGGGAGG	AATACTGAATACTGTGGCGT
QCR6	GCGTGCTCTGGATTCGATAA	CAGTGCATGAGGTGGAAGAA
CytB	CTTCTGGGTATTTGTGGCTA	ACGGTGAGTTTGGATGTTGA
ATP9（Reference gene）	AAACCCAGGACTAAGACCAC	ATGAATACAGCAATAGGAAG
