# Supplementary material for: Aptly chosen, effectively emphasizing the action and mechanism of antimycin A1
Source: Front Microbiol. 2024 Apr 3;15:1371850. doi: 10.3389/fmicb.2024.1371850 (PMC11021728; doi:10.3389/fmicb.2024.1371850)
Supplement: Supplementary file 4 [file Data_Sheet_4.PDF]

TABLE S2 Result from Pathway Analysis

Pathway Name	Match Status	p	-log(p)	Holm p	FDR	Impact	Details Show
Phenylalanine, tyrosine and tryptophan biosynthesis	9/21	3.8902E-8	7.41	2.8398E-6	2.8398E-6	0.33181	KEGG
Valine, leucine and isoleucine biosynthesis	8/20	4.8212E-7	6.3168	3.4713E-5	1.7597E-5	0.31595	KEGG
Cysteine and methionine metabolism	9/41	2.503E-5	4.6015	0.0017772	5.0449E-4	0.18176	KEGG
Glycine, serine and threonine metabolism	8/32	2.7643E-5	4.5584	0.001935	5.0449E-4	0.24108	KEGG
Aminoacyl-tRNA biosynthesis	9/46	6.7379E-5	4.1715	0.0046492	9.8374E-4	0.0	KEGG
Phenylalanine metabolism	4/7	9.3869E-5	4.0275	0.0063831	0.0011421	0.6	KEGG
Valine, leucine and isoleucine degradation	5/18	6.4628E-4	3.1896	0.0433	0.0067397	0.625	KEGG
Citrate cycle (TCA cycle)	4/20	0.0086461	2.0632	0.57064	0.078896	0.14556	KEGG
Alanine, aspartate and glutamate metabolism	4/22	0.012264	1.9114	0.79717	0.099476	0.1295	KEGG
Pyruvate metabolism	4/23	0.01439	1.8419	0.92099	0.10392	0.49237	KEGG
Sulfur metabolism	3/13	0.01566	1.8052	0.98656	0.10392	0.06169	KEGG
Butanoate metabolism	3/14	0.019343	1.7135	1.0	0.11767	0.0	KEGG
Glyoxylate and dicarboxylate metabolism	4/26	0.022129	1.655	1.0	0.12426	0.07831	KEGG
Lysine biosynthesis	3/16	0.028032	1.5523	1.0	0.14617	0.0	KEGG
Propanoate metabolism	3/19	0.044363	1.353	1.0	0.2159	0.04762	KEGG
