# Supplementary material for: Aptly chosen, effectively emphasizing the action and mechanism of antimycin A1
Source: Front Microbiol. 2024 Apr 3;15:1371850. doi: 10.3389/fmicb.2024.1371850 (PMC11021728; doi:10.3389/fmicb.2024.1371850)
Supplement: Supplementary file 5 [file Data_Sheet_5.PDF]

TABLE S3

The conversion results

	Query	Match	HMDB	PubChem	KEGG
1	C06002	(S)-Methylmalonic acid semialdehyde	HMDB0002217	5462303	C06002
2	C00024	Acetyl-CoA	HMDB0001206	444493	C00024
3	C00183	L-Valine	HMDB0000883	6287	C00183
4	C00123	L-Leucine	HMDB0000687	6106	C00123
5	C00407	L-Isoleucine	HMDB0000172	6306	C00407
6	C00021	S-Adenosylhomocysteine	HMDB0000939	439155	C00021
7	C00263	L-Homoserine	HMDB0000719	12647	C00263
8	C00073	L-Methionine	HMDB0000696	6137	C00073
9	C00491	L-Cystine	HMDB0000192	67678	C00491
10	C00979	O-Acetylserine	HMDB0003011	99478	C00979
11	C01234	1-Aminocyclopropanecarboxylic acid	HMDB0036458	535	C01234
12	C00049	L-Aspartic acid	HMDB0000191	5960	C00049
13	C00197	3-Phosphoglyceric acid	HMDB0060180	439183	C00197
14	C00170	5'-Methylthioadenosine	HMDB0001173	439176	C00170
15	C00022	Pyruvic acid	HMDB0000243	1060	C00022
16	C00051	Glutathione	HMDB0062697	745	C00051
17	C03089	5-Methylthioribose	HMDB0001087	439904	C03089
18	C00550	SM(d18:1/18:0)	HMDB0001348	5283588	C00550
19	C01120	Sphinganine 1-phosphate	HMDB0001383	644260	C01120
20	C02686	Galactosylceramide (d18:1/16:0)	HMDB0010708	53480652	C02686
21	C17235	L-Homophenylalanine	-	96023643	C17235
22	C00079	L-Phenylalanine	HMDB0000159	6140	C00079
23	C00463	Indole	HMDB0000738	798	C00463
24	C00078	L-Tryptophan	HMDB0000929	6305	C00078
25	C00082	L-Tyrosine	HMDB0000158	6057	C00082
26	C02637	3-Dehydroshikimate	METPA0312	-	C02637
27	C00166	Phenylpyruvic acid	HMDB0000205	997	C00166
28	C00254	Prephenate	HMDB0012283	1028	C00254
29	C00108	2-Aminobenzoic acid	HMDB0001123	227	C00108
30	C00587	3-Hydroxybenzoic acid	HMDB0002466	7420	C00587
31	C00826	L-Arogenate	METPA0086	-	C00826
32	C03506	Indoleglycerol phosphate	METPA0401	-	C03506
33	C00546	Pyruvaldehyde	HMDB0001167	880	C00546
34	C00188	L-Threonine	HMDB0000167	6288	C00188
35	C00114	Choline	HMDB0000097	305	C00114
36	C00300	Creatine	HMDB0000064	586	C00300
37	C05519	L-Allothreonine	HMDB0004041	99289	C05519

38	C00258	Glyceric acid	HMDB0000139	439194	C00258
39	C06006	(S)-2-Aceto-2-hydroxybutanoic acid	HMDB0006900	440875	C06006
40	C02631	Isopropylmaleate	HMDB0012241	5280533	C02631
41	C04411	3-Isopropylmalate	HMDB0012156	5462261	C04411
42	C05332	Phenylethylamine	HMDB0012275	1001	C05332
43	C05853	2-Phenylethanol	HMDB0033944	6054	C05853
44	C02265	D-Phenylalanine	METPA0264	-	C02265
45	C05593	3-Hydroxyphenylacetic acid	HMDB0000440	12122	C05593
46	C04148	Alpha-N-Phenylacetyl-L-glutamine	HMDB0006344	92258	C04148
47	C00122	Fumaric acid	HMDB0000134	444972	C00122
48	C00042	Succinic acid	HMDB0000254	1110	C00042
49	C07086	Phenylacetic acid	HMDB0000209	999	C07086
