# Supplementary figures and images for: Aptly chosen, effectively emphasizing the action and mechanism of antimycin A1
Source: Front Microbiol. 2024 Apr 3;15:1371850. doi: 10.3389/fmicb.2024.1371850 (PMC11021728; doi:10.3389/fmicb.2024.1371850)

**TABLE S1** Result from Pathway Analysis


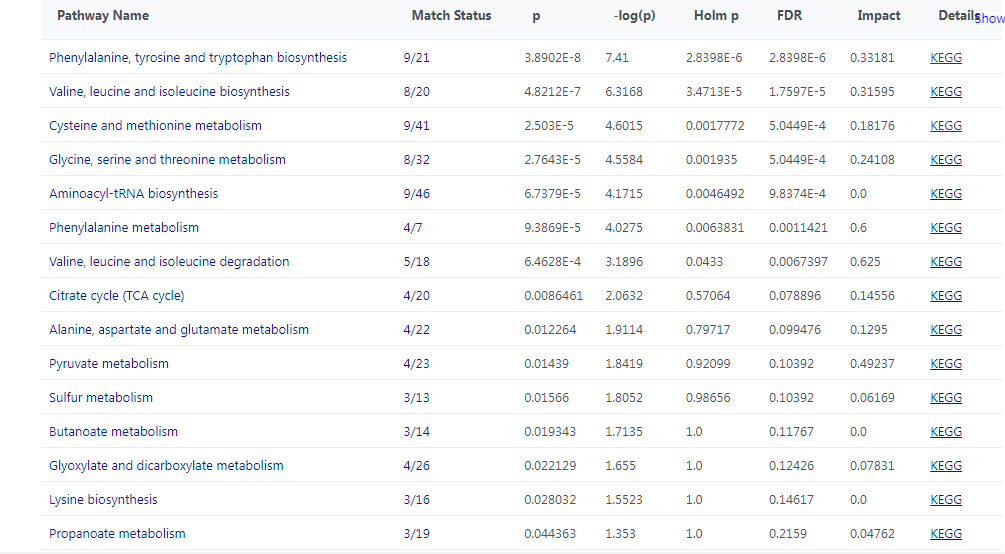

Supplement: Supplementary file 6 [file Table_1.DOC]
