# Supplementary material for: Aptly chosen, effectively emphasizing the action and mechanism of antimycin A1
Source: Front Microbiol. 2024 Apr 3;15:1371850. doi: 10.3389/fmicb.2024.1371850 (PMC11021728; doi:10.3389/fmicb.2024.1371850)
Supplement: Supplementary file 7 [file Table_2.DOC]

| Query Match HMDB PubChem KEGG | | | | | |
| --- | --- | --- | --- | --- | --- |
| 1 | C06002 | (S)-Methylmalonic acid semialdehyde | [HMDB0002217](http://www.hmdb.ca/metabolites/HMDB0002217) | [5462303](http://pubchem.ncbi.nlm.nih.gov/summary/summary.cgi?cid=5462303) | C06002 |
| 2 | C00024 | Acetyl-CoA | [HMDB0001206](http://www.hmdb.ca/metabolites/HMDB0001206) | [444493](http://pubchem.ncbi.nlm.nih.gov/summary/summary.cgi?cid=444493) | C00024 |
| 3 | C00183 | L-Valine | [HMDB0000883](http://www.hmdb.ca/metabolites/HMDB0000883) | [6287](http://pubchem.ncbi.nlm.nih.gov/summary/summary.cgi?cid=6287) | C00183 |
| 4 | C00123 | L-Leucine | [HMDB0000687](http://www.hmdb.ca/metabolites/HMDB0000687) | [6106](http://pubchem.ncbi.nlm.nih.gov/summary/summary.cgi?cid=6106) | C00123 |
| 5 | C00407 | L-Isoleucine | [HMDB0000172](http://www.hmdb.ca/metabolites/HMDB0000172) | [6306](http://pubchem.ncbi.nlm.nih.gov/summary/summary.cgi?cid=6306) | C00407 |
| 6 | C00021 | S-Adenosylhomocysteine | [HMDB0000939](http://www.hmdb.ca/metabolites/HMDB0000939) | [439155](http://pubchem.ncbi.nlm.nih.gov/summary/summary.cgi?cid=439155) | C00021 |
| 7 | C00263 | L-Homoserine | [HMDB0000719](http://www.hmdb.ca/metabolites/HMDB0000719) | [12647](http://pubchem.ncbi.nlm.nih.gov/summary/summary.cgi?cid=12647) | C00263 |
| 8 | C00073 | L-Methionine | [HMDB0000696](http://www.hmdb.ca/metabolites/HMDB0000696) | [6137](http://pubchem.ncbi.nlm.nih.gov/summary/summary.cgi?cid=6137) | C00073 |
| 9 | C00491 | L-Cystine | [HMDB0000192](http://www.hmdb.ca/metabolites/HMDB0000192) | [67678](http://pubchem.ncbi.nlm.nih.gov/summary/summary.cgi?cid=67678) | C00491 |
| 10 | C00979 | O-Acetylserine | [HMDB0003011](http://www.hmdb.ca/metabolites/HMDB0003011) | [99478](http://pubchem.ncbi.nlm.nih.gov/summary/summary.cgi?cid=99478) | C00979 |
| 11 | C01234 | 1-Aminocyclopropanecarboxylic acid | [HMDB0036458](http://www.hmdb.ca/metabolites/HMDB0036458) | [535](http://pubchem.ncbi.nlm.nih.gov/summary/summary.cgi?cid=535) | C01234 |
| 12 | C00049 | L-Aspartic acid | [HMDB0000191](http://www.hmdb.ca/metabolites/HMDB0000191) | [5960](http://pubchem.ncbi.nlm.nih.gov/summary/summary.cgi?cid=5960) | C00049 |
| 13 | C00197 | 3-Phosphoglyceric acid | [HMDB0060180](http://www.hmdb.ca/metabolites/HMDB0060180) | [439183](http://pubchem.ncbi.nlm.nih.gov/summary/summary.cgi?cid=439183) | C00197 |
| 14 | C00170 | 5'-Methylthioadenosine | [HMDB0001173](http://www.hmdb.ca/metabolites/HMDB0001173) | [439176](http://pubchem.ncbi.nlm.nih.gov/summary/summary.cgi?cid=439176) | C00170 |
| 15 | C00022 | Pyruvic acid | [HMDB0000243](http://www.hmdb.ca/metabolites/HMDB0000243) | [1060](http://pubchem.ncbi.nlm.nih.gov/summary/summary.cgi?cid=1060) | C00022 |
| 16 | C00051 | Glutathione | [HMDB0062697](http://www.hmdb.ca/metabolites/HMDB0062697) | [745](http://pubchem.ncbi.nlm.nih.gov/summary/summary.cgi?cid=745) | C00051 |
| 17 | C03089 | 5-Methylthioribose | [HMDB0001087](http://www.hmdb.ca/metabolites/HMDB0001087) | [439904](http://pubchem.ncbi.nlm.nih.gov/summary/summary.cgi?cid=439904) | C03089 |
| 18 | C00550 | SM(d18:1/18:0) | [HMDB0001348](http://www.hmdb.ca/metabolites/HMDB0001348) | [5283588](http://pubchem.ncbi.nlm.nih.gov/summary/summary.cgi?cid=5283588) | C00550 |
| 19 | C01120 | Sphinganine 1-phosphate | [HMDB0001383](http://www.hmdb.ca/metabolites/HMDB0001383) | [644260](http://pubchem.ncbi.nlm.nih.gov/summary/summary.cgi?cid=644260) | C01120 |
| 20 | C02686 | Galactosylceramide (d18:1/16:0) | [HMDB0010708](http://www.hmdb.ca/metabolites/HMDB0010708) | [53480652](http://pubchem.ncbi.nlm.nih.gov/summary/summary.cgi?cid=53480652) | C02686 |
| 21 | C17235 | L-Homophenylalanine | - | [96023643](http://pubchem.ncbi.nlm.nih.gov/summary/summary.cgi?cid=96023643) | C17235 |
| 22 | C00079 | L-Phenylalanine | [HMDB0000159](http://www.hmdb.ca/metabolites/HMDB0000159) | [6140](http://pubchem.ncbi.nlm.nih.gov/summary/summary.cgi?cid=6140) | C00079 |
| 23 | C00463 | Indole | [HMDB0000738](http://www.hmdb.ca/metabolites/HMDB0000738) | [798](http://pubchem.ncbi.nlm.nih.gov/summary/summary.cgi?cid=798) | C00463 |
| 24 | C00078 | L-Tryptophan | [HMDB0000929](http://www.hmdb.ca/metabolites/HMDB0000929) | [6305](http://pubchem.ncbi.nlm.nih.gov/summary/summary.cgi?cid=6305) | C00078 |
| 25 | C00082 | L-Tyrosine | [HMDB0000158](http://www.hmdb.ca/metabolites/HMDB0000158) | [6057](http://pubchem.ncbi.nlm.nih.gov/summary/summary.cgi?cid=6057) | C00082 |
| 26 | C02637 | 3-Dehydroshikimate | [METPA0312](http://www.hmdb.ca/metabolites/METPA0312) | - | C02637 |
| 27 | C00166 | Phenylpyruvic acid | [HMDB0000205](http://www.hmdb.ca/metabolites/HMDB0000205) | [997](http://pubchem.ncbi.nlm.nih.gov/summary/summary.cgi?cid=997) | C00166 |
| 28 | C00254 | Prephenate | [HMDB0012283](http://www.hmdb.ca/metabolites/HMDB0012283) | [1028](http://pubchem.ncbi.nlm.nih.gov/summary/summary.cgi?cid=1028) | C00254 |
| 29 | C00108 | 2-Aminobenzoic acid | [HMDB0001123](http://www.hmdb.ca/metabolites/HMDB0001123) | [227](http://pubchem.ncbi.nlm.nih.gov/summary/summary.cgi?cid=227) | C00108 |
| 30 | C00587 | 3-Hydroxybenzoic acid | [HMDB0002466](http://www.hmdb.ca/metabolites/HMDB0002466) | [7420](http://pubchem.ncbi.nlm.nih.gov/summary/summary.cgi?cid=7420) | C00587 |
| 31 | C00826 | L-Arogenate | [METPA0086](http://www.hmdb.ca/metabolites/METPA0086) | - | C00826 |
| 32 | C03506 | Indoleglycerol phosphate | [METPA0401](http://www.hmdb.ca/metabolites/METPA0401) | - | C03506 |
| 33 | C00546 | Pyruvaldehyde | [HMDB0001167](http://www.hmdb.ca/metabolites/HMDB0001167) | [880](http://pubchem.ncbi.nlm.nih.gov/summary/summary.cgi?cid=880) | C00546 |
| 34 | C00188 | L-Threonine | [HMDB0000167](http://www.hmdb.ca/metabolites/HMDB0000167) | [6288](http://pubchem.ncbi.nlm.nih.gov/summary/summary.cgi?cid=6288) | C00188 |
| 35 | C00114 | Choline | [HMDB0000097](http://www.hmdb.ca/metabolites/HMDB0000097) | [305](http://pubchem.ncbi.nlm.nih.gov/summary/summary.cgi?cid=305) | C00114 |
| 36 | C00300 | Creatine | [HMDB0000064](http://www.hmdb.ca/metabolites/HMDB0000064) | [586](http://pubchem.ncbi.nlm.nih.gov/summary/summary.cgi?cid=586) | C00300 |
| 37 | C05519 | L-Allothreonine | [HMDB0004041](http://www.hmdb.ca/metabolites/HMDB0004041) | [99289](http://pubchem.ncbi.nlm.nih.gov/summary/summary.cgi?cid=99289) | C05519 |
| 38 | C00258 | Glyceric acid | [HMDB0000139](http://www.hmdb.ca/metabolites/HMDB0000139) | [439194](http://pubchem.ncbi.nlm.nih.gov/summary/summary.cgi?cid=439194) | C00258 |
| 39 | C06006 | (S)-2-Aceto-2-hydroxybutanoic acid | [HMDB0006900](http://www.hmdb.ca/metabolites/HMDB0006900) | [440875](http://pubchem.ncbi.nlm.nih.gov/summary/summary.cgi?cid=440875) | C06006 |
| 40 | C02631 | Isopropylmaleate | [HMDB0012241](http://www.hmdb.ca/metabolites/HMDB0012241) | [5280533](http://pubchem.ncbi.nlm.nih.gov/summary/summary.cgi?cid=5280533) | C02631 |
| 41 | C04411 | 3-Isopropylmalate | [HMDB0012156](http://www.hmdb.ca/metabolites/HMDB0012156) | [5462261](http://pubchem.ncbi.nlm.nih.gov/summary/summary.cgi?cid=5462261) | C04411 |
| 42 | C05332 | Phenylethylamine | [HMDB0012275](http://www.hmdb.ca/metabolites/HMDB0012275) | [1001](http://pubchem.ncbi.nlm.nih.gov/summary/summary.cgi?cid=1001) | C05332 |
| 43 | C05853 | 2-Phenylethanol | [HMDB0033944](http://www.hmdb.ca/metabolites/HMDB0033944) | [6054](http://pubchem.ncbi.nlm.nih.gov/summary/summary.cgi?cid=6054) | C05853 |
| 44 | C02265 | D-Phenylalanine | [METPA0264](http://www.hmdb.ca/metabolites/METPA0264) | - | C02265 |
| 45 | C05593 | 3-Hydroxyphenylacetic acid | [HMDB0000440](http://www.hmdb.ca/metabolites/HMDB0000440) | [12122](http://pubchem.ncbi.nlm.nih.gov/summary/summary.cgi?cid=12122) | C05593 |
| 46 | C04148 | Alpha-N-Phenylacetyl-L-glutamine | [HMDB0006344](http://www.hmdb.ca/metabolites/HMDB0006344) | [92258](http://pubchem.ncbi.nlm.nih.gov/summary/summary.cgi?cid=92258) | C04148 |
| 47 | C00122 | Fumaric acid | [HMDB0000134](http://www.hmdb.ca/metabolites/HMDB0000134) | [444972](http://pubchem.ncbi.nlm.nih.gov/summary/summary.cgi?cid=444972) | C00122 |
| 48 | C00042 | Succinic acid | [HMDB0000254](http://www.hmdb.ca/metabolites/HMDB0000254) | [1110](http://pubchem.ncbi.nlm.nih.gov/summary/summary.cgi?cid=1110) | C00042 |
| 49 | C07086 | Phenylacetic acid | [HMDB0000209](http://www.hmdb.ca/metabolites/HMDB0000209) | [999](http://pubchem.ncbi.nlm.nih.gov/summary/summary.cgi?cid=999) | C07086 |
